# The Effect of Age-Related Macular Degeneration on Components of Face Perception

**DOI:** 10.1167/iovs.61.6.38

**Published:** 2020-06-16

**Authors:** Andrew J. Logan, Gael E. Gordon, Gunter Loffler

**Affiliations:** 1 Department of Vision Sciences, Glasgow Caledonian University, Glasgow, United Kingdom; 2 School of Optometry and Vision Science, University of Bradford, Bradford, United Kingdom

**Keywords:** face perception, psychophysics, age-related macular degeneration, face features

## Abstract

**Purpose:**

Patients with age-related macular degeneration (AMD) experience difficulty with discriminating between faces. We aimed to use a new clinical test to quantify the impact of AMD on face perception and to determine the specific aspects that are affected.

**Methods:**

The Caledonian face test uses an adaptive procedure to measure face discrimination thresholds: the minimum difference required between faces for reliable discrimination. Discrimination thresholds were measured for full-faces, external features (head-shape and hairline), internal features (nose, mouth, eyes, and eyebrows) and shapes (non-face task). Participants were 20 patients with dry AMD (logMAR VA = 0.14 to 0.62), 20 patients with wet AMD (0.10 to 0.60), and 20 age-matched control subjects (−0.18 to +0.06).

**Results:**

Relative to controls, full-face discrimination thresholds were, on average, 1.76 and 1.73 times poorer in participants with dry and wet AMD, respectively. AMD also reduced sensitivity to face features, but discrimination of the internal, relative to external, features was disproportionately impaired. Both distance VA and contrast sensitivity were significant independent predictors of full-face discrimination thresholds (*R*^2^ = 0.66). Sensitivity to full-faces declined by a factor of approximately 1.19 per 0.1 logMAR reduction in VA.

**Conclusions:**

Both dry and wet AMD significantly reduce sensitivity to full-faces and their component parts to similar extents. Distance VA and contrast sensitivity are closely associated with face discrimination sensitivity. These results quantify the extent of sensitivity impairment in patients with AMD and predict particular difficulty in everyday tasks that rely on internal feature information, including recognition of familiar faces and facial expressions.

Age-related macular degeneration (AMD) is a chronic and progressive disease of the central retina^1^ and a leading cause of visual impairment among older adults in Western Europe and the USA.^2^^,^^3^ It has been estimated that late AMD affects 4.8% of adults aged over 65 years old in the United Kingdom and, because of an aging population, the number of affected individuals will increase considerably over time.^4^ AMD is characterized by structural changes within the macular region of the retina that disrupt the processing of visual information.^5^ AMD significantly impairs aspects of low-level vision (e.g., visual acuity (VA) and contrast sensitivity)^6^ which, in turn, impacts on more complex visual functions.^7^^,^^8^ As a result, patients with AMD report difficulties with everyday tasks such as reading, mobility, and face perception.^9^^–^^11^

Faces are complex visual objects that contain a wealth of information. A brief glimpse of a face is sufficient for the visual system to accurately discriminate between individual identities.^12^ Because all faces are based on the same template (two eyes, above a nose, above a mouth), discriminating between identities requires sensitivity to subtle differences in the shape and position of the face features. Processing this information relies on the high resolution of central vision.^7^ Accordingly, patients with AMD have identified face perception as a task with which they experience particular difficulty.^11^^,^^13^^,^^14^ This impairment has a negative impact on quality of life^9^^,^^10^ and has been identified by patients as a priority for improvement.^15^

Although measurements of low-level vision, such as VA, are related to face perception ability in patients with AMD,^14^^,^^16^^,^^17^ these measures do not fully capture the degree of functional disability experienced by patients.^13^^,^^18^^,^^19^ The variability in face discrimination ability in patients with AMD is significantly greater than that predicted by measurements of low-level vision.^14^^,^^16^ This may be a consequence of the hierarchical nature of visual processing; face discrimination relies on both low-level vision and more complex processing mechanisms, such as those that are specialized for faces.^7^ As a result, measurements of low-level vision alone may not provide a reliable indication of difficulties with face processing experienced by patients with AMD.

A number of specific face perception tests have been previously used with patients with AMD. Several of these paradigms, however, are limited by restricted testing ranges.^16^^,^^20^^,^^21^ For example, although Barnes and colleagues^16^ found that AMD participants demonstrated an impairment of face matching, a number of control participants scored 100% (i.e., a ceiling effect). Tests that are limited by ceiling effects may underestimate the impact of AMD on face perception.

Other tests used to measure face perception ability in AMD required participants to learn and recognize individual face identities.^17^ This paradigm cannot separate specific impairments of vision from more general differences in cognition (e.g., memory, familiarity determination). Similarly, other tests ask participants to identify photographs of celebrities.^14^^,^^22^ Performance on these tests is dependent on familiarity with specific faces.

We have recently designed a new test of face discrimination^23^ that provides a rapid (average test time is approximately four minutes) yet repeatable and sensitive quantification of face discrimination sensitivity. The test makes no memory demands, and the range is essentially unlimited (i.e., no ceiling or floor effects). This study aimed to use this test to quantify the effect of AMD on visual sensitivity to face information.

There are two types of AMD: nonexudative (dry) and exudative (wet). Whereas the end-point of dry AMD is geographic atrophy of the photoreceptors and retinal pigment epithelium, wet AMD, on the other hand, is characterized by choroidal neovascularization, hemorrhaging, and subsequent scarring.^24^ It has been reported that these differences in pathophysiology result in significant differences in the effects of dry and wet AMD on specific visual functions including reading speed,^25^ perception of distortion^26^ and contrast sensitivity.^27^ Although previous studies have included participants with both dry and wet AMD,^21^^,^^28^ to our knowledge, there has been no comparison of the effect of the two types of AMD on face discrimination ability.

Within the wealth of information in face stimuli, a distinction is commonly made between the internal (nose, eyes, mouth, and eyebrows) and external (head-shape and hairline) face features. In typical participants, the external features make a disproportionate contribution to unfamiliar face discrimination^29^^,^^30^; the internal features are particularly important for familiar face recognition.^31^^–^^33^ Impairments of face perception are associated with significant changes in face processing strategies. For example, it has been reported that, unlike typical participants, some patients with prosopagnosia (a specific impairment of face perception) rely on the external features to identify both familiar and unfamiliar faces.^34^

When viewing a familiar face, patients with AMD make more fixations toward the external features, and look at the internal features considerably less, than participants with healthy vision.^28^ This atypical pattern of eye movements suggests that patients with AMD may use a modified face processing strategy that may indicate that AMD does not impair sensitivity to all face features equally. Sensitivity to the internal and external face features in AMD, however, remains to be quantified.

Discriminating between groups of internal face features is dependent on the resolution of idiosyncratic differences in the shapes and positions of individual features (e.g., interocular separation, lip thickness). Processing this information relies on the high resolution of central vision.^7^ External features, on the other hand, can be distinguished based on differences in global shapes that are more spatially expansive than the internal features. Accordingly, one possible outcome is that compromised high-resolution central vision in AMD disproportionately reduces sensitivity to the internal face features. In support of this premise, presenting internal face features in peripheral, relative to central, vision in typical participants reduces sensitivity to a considerably greater extent than for the external features.^35^ This result suggests that impoverished spatial resolution—such as that found in AMD and in normal peripheral vision—is more detrimental to the processing of internal, relative to external, features.

On the other hand, Wang and colleagues^36^ identified a significant deficit of global shape discrimination sensitivity in patients with early AMD, despite relatively intact low-level vision (poorest VA = 0.40 LogMAR).^8^ The shapes used by Wang and colleagues^36^ were radial frequency patterns; closed contours that can be used to describe biological shapes, including human heads.^37^ This result raises the possibility that sensitivity to the external face features (e.g., head-shape) may also be vulnerable to the effects of AMD. This study aimed to investigate the extent and nature of face processing in AMD by quantitatively comparing the impact of both dry and wet AMD on sensitivity to full faces and internal and external face features.

## Methods

### Synthetic Faces

**Figure 1.**
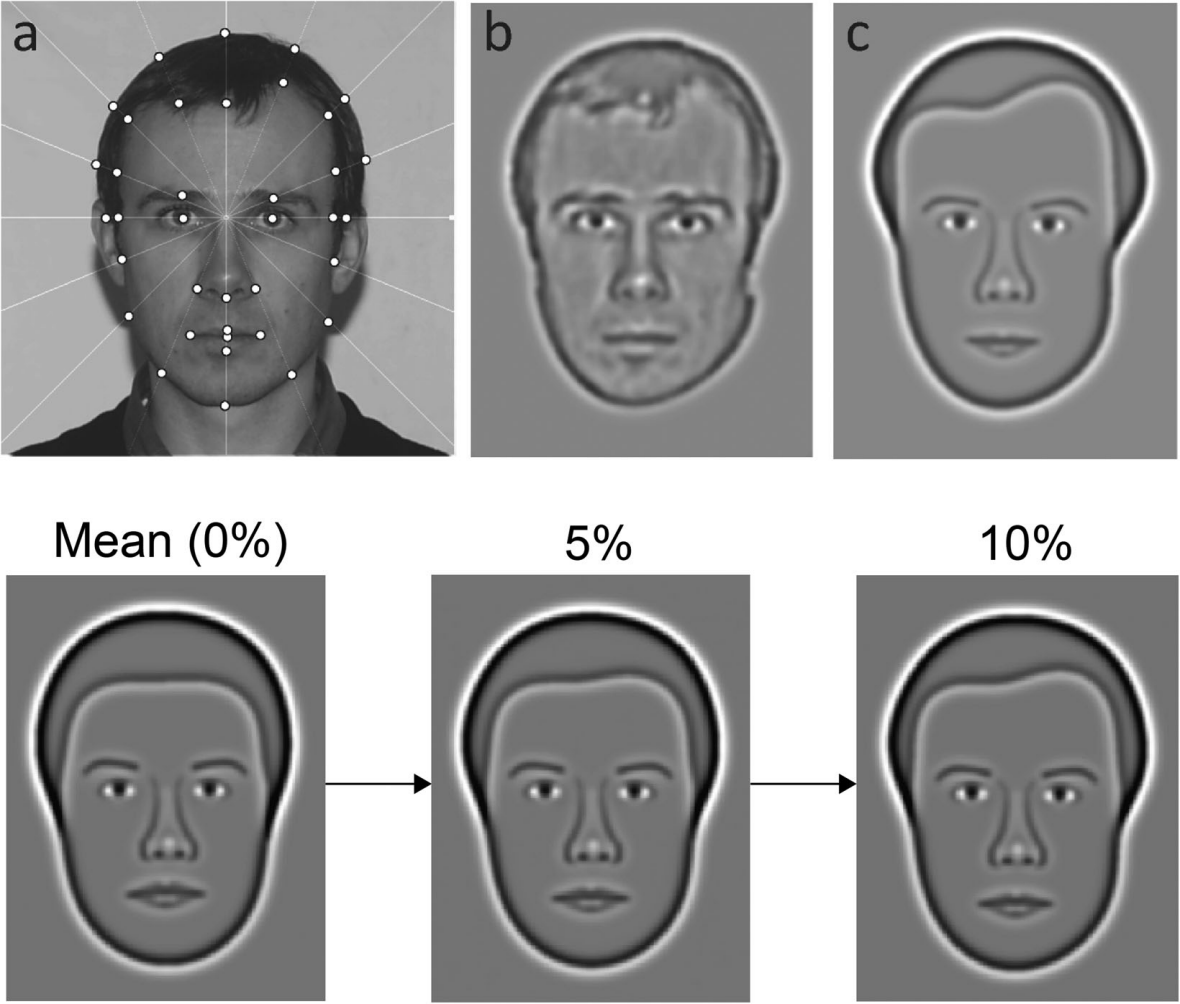
Synthetic faces. *Top:* (**a**) Grayscale photograph superimposed with polar coordinate grid centered on the bridge of the nose. The head-shape was measured at 16 locations (*white dots*) around the external contour, angularly positioned at equal intervals of 22.5°. The polar co-ordinates of 14 of the measured points were used to define seven radial frequencies (RFs) to describe the subject's head-shape. RF patterns^36^ are circular contours with sinusoidally-modulated radii that can be used to describe a range of natural shapes including fruits and head shapes. A further nine points were used to define four RFs that captured the shape of the subject's hairline. All RFs were defined relative to the mean head radius of all synthetic faces of the subject's sex. The location and shape of the internal face features were also digitized. In sum, the face is described by 37 measurements. (**b**) Photograph filtered with a 2.0-octave bandwidth DOG filter with peak spatial frequency of 10 cycles/face width for comparison with corresponding synthetic face (**c**). *Bottom*: synthetic faces were adjusted by manipulating how much they differ from the mean face (left). Increasing face difference results in individual faces becoming progressively more dissimilar (from middle to right) to the mean face. Face difference is expressed as a percentage of mean head radius and quantifies the total geometric variation between any specified face and the mean face. Typical observers can discriminate a face from the mean at about 5% face difference.

Synthetic faces^37^ capture the major geometric face information from gray-scale face photographs with neutral expressions. Some aspects of the following description of our stimuli and procedure have been published elsewhere.^23^^,^^30^ A polar coordinate grid was superimposed on the face photograph, centered on the bridge of the subject's nose (Fig. 1a). The external contour of the subject's head was interpolated from 16 equally spaced measurements; the hairline from 9 further points. The internal features were defined by 14 additional measurements. While the position of all features was idiosyncratic, the shape of the eyes and eyebrows was generic. Individuating information was contained within variations in horizontal and vertical eye position, in addition to the height of the eyebrows, defined relative to the center of the eyes. The mouth and nose shapes were derived from generic forms that were altered in terms of length and width based on individual face measurements. In sum, each synthetic face is defined by 37 parameters and represented by a 37-dimensional vector.

The images were subsequently band-pass filtered (circular DOG filter with a bandwidth of 2.0 octaves) at the optimal spatial frequency for face identification (10 cycles/face-width; Fig. 1b).^38^ The resulting faces accentuate geometric information in the most important frequency band while omitting high spatial frequency cues (e.g., hair texture, skin wrinkles) which contribute little to face identification.^39^ This filtering gives synthetic faces a distinct advantage over unfiltered, broadband stimuli typically used to measure face perception. Specifically, the filtering of high spatial frequency detail enables synthetic faces to achieve a degree of independence from small variations in resolution ability. As a result, the Caledonian face test is robust to small variations in visual acuity.^23^

A mean face was produced by averaging each of the 37 dimensions of all synthetic faces of the same gender. All faces were expressed relative to the gender-appropriate mean face, which served as the origin of a multidimensional face space. Within this framework, synthetic faces can be morphed to have any defined geometric difference from the mean face (Fig. 1, bottom). This value, expressed as a percentage of the mean head size, quantifies the distinctiveness of individual faces. Previous studies have shown that this correlates closely with discrimination sensitivity.^37^ All synthetic faces were scaled to the same size. At the test distance of 1.2 m, each face subtended 5.5° of visual angle in height.

### The Caledonian Face Test

The Caledonian face test is a computer-based odd-one-out task which provides a rapid quantification of the face discrimination threshold (i.e., the minimum difference required between faces for reliable discrimination).^23^

**Figure 2.**
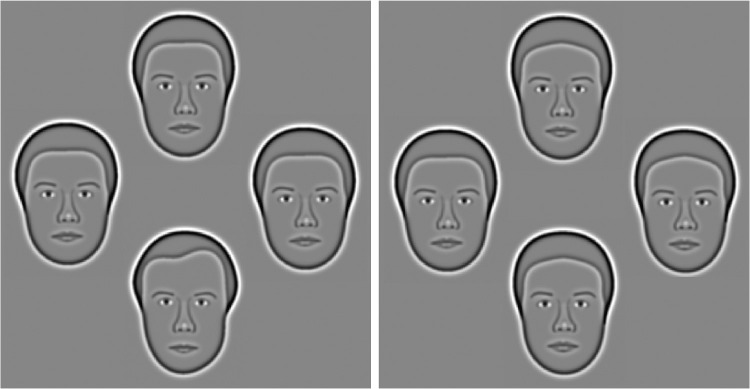
The Caledonian face test. Participants were presented with four faces arranged in a diamond configuration and were asked to indicate the “odd” face that differed from the others. *Left*: suprathreshold trial for most participants (target face differs from mean face by 10%). The target (odd one) is at the bottom. Right: difficult trial, approximately at threshold for a typical participant (5%). Target is to the left.

Participants were shown four faces in a diamond configuration (Fig. 2). Three of the faces were identical (distracters) while one face (target) was morphed to differ from the distracters by a specified amount. Participants were asked to respond by indicating the odd-one-out via computer mouse click and guess when uncertain. Viewing time was unlimited. The mean face, which featured on every trial, was randomly assigned as the target face on 50% of trials. The identity of the other face was randomly selected from a large database (40 male, 40 female). Face gender was randomly selected for each trial; the gender of the mean face was matched to that of the non-mean face.

The magnitude of the difference between the faces on each trial was controlled by a QUEST adaptive procedure.^40^ This highly efficient algorithm adjusts the task difficulty to concentrate testing around the participant's face discrimination threshold. QUEST utilizes a maximum likelihood procedure to produce a threshold estimate after each trial based on all responses made from the beginning of the test run.

To maintain participant engagement, dummy trials (face difference set to 3 times current threshold estimate) were included on every 7^th^ trial. Following earlier validation work,^23^ the face discrimination threshold was defined as the best estimate of threshold at the conclusion of a 30-trial-run.

### Apparatus

The study was carried out with binocular viewing under an ambient illumination of 75 cd m^2^. Participants were seated at 1.2m from an HP P1230 monitor (1024 X 768 at 85 Hz; Hewlett-Packard, Palo Alto, CA, USA) of 64 cd m^2^ mean luminance that was controlled by an Apple Mac Pro computer. The color look-up table was defined to maximize contrast linearity of the monitor. The Caledonian face test was written in Matlab (www.mathworks.com) and includes routines from the Psychtoolbox extension.^41^^,^^42^

### Participants

Forty naïve patients with AMD (20 non-exudative, 20 exudative) took part in the study (Table 1). Patients were recruited from the University of Bradford's eye clinic and with the assistance of the Macular Society, a charity for patients with central vision loss. All patients had been diagnosed with bilateral AMD by an ophthalmologist and had no history of other ocular diseases (e.g., cataract, glaucoma), amblyopia or strabismus. An optometrist examined the eyes of all patients to screen for significant crystalline lens opacities and to document the retinal signs of AMD. Patients were divided into two categories: those with nonexudative (dry) AMD only and those with AMD, which included exudative (wet) changes in either eye. Binocular VA (LogMAR) ranged from 0.14 to 0.62 and 0.10 to 0.60 in the dry AMD and wet AMD groups respectively (see Table 1).

**Table 1.** Participant Information

	Controls	Dry AMD	Wet AMD
*N*	20	20	20
*N* male	8	8	7
Mean age (range)	76.7 (67–88)	76.4 (66–87)	76.9 (67–86)
Mean VA (Binocular) LogMAR (range)	−0.03 (−0.18 to 0.06)	0.28 (0.14 to 0.62)	0.30 (0.10 to 0.60)
Mean VA (Better eye) LogMAR (range)	−0.02 (−0.18 to 0.06)	0.30 (0.12 to 0.62)	0.28 (0.04 to 0.60)
Mean VA (Poorer eye) LogMAR (range)	−0.01 (−0.16 to 0.06)	0.36 (0.16 to 1.00)	0.42 (0.24 to 1.00)
Mean binocular acuity gain (ratio)^*^ (range)	1.01 (0.25 to 2)	1.06 (0.86 to 1.25)	0.93 (0.40 to 1.13)
Mean CS (Binocular) Log units (range)	1.76 (1.65 to 1.95)	1.26 (0.75 to 1.65)	1.18 (0.75 to 1.65)
Mean MoCA Score (SD)	28.9 (1.0)	28.7 (1.0)	28.4 (1.0)

MoCA, Montreal Cognitive Assessment.

* Binocular acuity gain (ratio) calculated as VA (Better Eye)/VA (Binocular).

Twenty age-matched control participants with healthy vision were recruited from the University of Bradford's eye clinic. Record cards were screened for significant cataract, macular degeneration, raised intra-ocular pressure (>21 mm Hg) or visual field loss. All control participants were in good health with no history of ocular disease, and normal, or corrected-to-normal, vision (best-achievable binocular VA of at least 0.10 LogMAR and binocular contrast sensitivity of at least 1.65 log units). Participants with amblyopia (greater than one line difference in VA between the eyes), strabismus or refractive error of greater than ±6.00 DS or 2.50 DC were excluded.

Participants gave informed consent in accordance with the Declaration of Helsinki, as approved by the Committee for Ethics in Research of the University of Bradford. Participants received £10 as compensation for their time. The Montreal Cognitive Assessment (MoCA)^43^ was used to screen for cognitive impairment. All participants passed this test (i.e., scores exceeded 26 points out of a possible 30).

Optimal refractive correction was determined for each participant and, where required, provided by trial lenses mounted in a trial frame. Distance VA was measured with an Early Treatment Diabetic Retinopathy Study chart at 3 m. Contrast sensitivity was assessed with a Pelli-Robson test chart.^44^ Both charts were displayed at the luminance recommended by the manufacturers.

### Procedure

**Figure 3.**
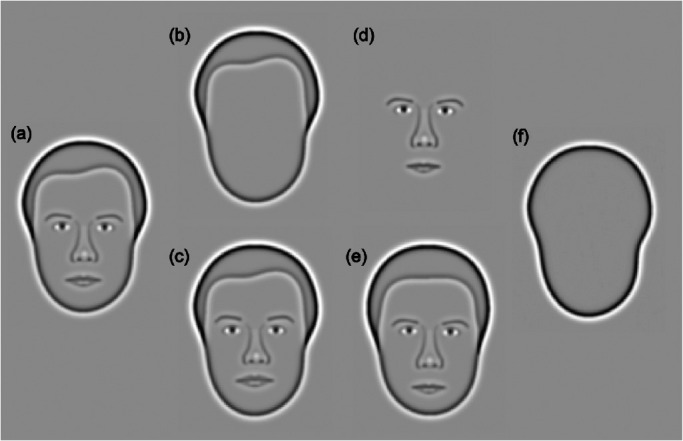
Face feature stimuli The Caledonian face test was administered under the following conditions: (**a**) full faces—all features vary by equivalent proportions, (**b**) isolated external features—only the head-shape and hairline are visible, (**c**) embedded external features—the same stimulus as (**b**), embedded within an otherwise fixed face context, (**d**) isolated internal features—only the eyes, nose, mouth, and eyebrows are visible, (**e**) embedded internal features—the same stimulus as (**d**), embedded within an otherwise fixed face context, (**f**) shapes.

After measurement of VA, CS, and cognition, participants completed one practice run of the Caledonian face test in which feedback was provided. The test was then used to measure discrimination thresholds for full-faces in which all of the features changed by equivalent proportions (Fig. 3a), and combinations of individual face features. Specifically, discrimination thresholds were measured for the external (head-shape and hairline) and internal (eyes, nose, mouth, and eyebrows) face features. Thresholds for these features were measured both in isolation (Figs. 3b, 3d) and embedded within a fixed face context (Figs. 3c, 3e). Isolated conditions presented individual features from the corresponding full face condition. For example, to create the “isolated external feature condition,” shown at 10% face difference, we extracted the head-shape and hairline from a full face shown at 10% face difference (see Fig. 3b).

Embedded conditions were used to measure discrimination thresholds for internal and external features while participants viewed whole faces, rather than isolated features. Only the features of interest varied between the target and distracters; all other features were identical. Accordingly, the features displayed within the embedded condition were the same as those shown within the associated isolated condition, with the addition of a task-irrelevant fixed face context. For example, in the embedded external feature condition, the difference between the target and distracter faces lies solely in the head-shape and hairline; the internal features were the same. Since the task-irrelevant features were identical across all options, faces in the embedded feature condition contained no more discrimination cues than the associated isolated feature condition.

Comparison of data for the isolated and embedded feature conditions provides insight into the strategies used to process combinations of face features. Specifically, similar thresholds for isolated and embedded features suggests that adults with healthy vision process external and internal face features independently.^45^ This study aimed to determine whether patients with AMD demonstrate the same qualitative pattern of face feature processing as healthy controls. Differences in thresholds for isolated and embedded features in patients with AMD would suggest that the disease leads to a qualitative change in this strategies utilized by the visual system to process face feature information. One possibility is that patients with AMD may demonstrate a fixed reliance on the external features that may appear less degraded, relative to the internal features, by low-level visual impairment. Such a qualitative change in processing strategy would be revealed by comparing thresholds for the isolated and embedded internal feature condition.

Finally, the Caledonian face test was adapted to measure discrimination thresholds for shapes, which served as a non-face control object (Fig. 3f). This condition provided a measure of visual function which is neither low-level (such as visual acuity or contrast sensitivity) nor specific to face perception. One possible outcome is that the data will suggest that AMD disproportionately impairs face perception, relative to other visual functions. If, on the other hand, our data indicate that AMD impairs shape discrimination and face perception to the same extent, this would suggest that the impact of AMD on higher-level visual functions can be attributed to impoverished low-level visual input.

The order of testing was randomized. Participants were not informed of the condition being tested and were always instructed to identify the odd-one-out.

### Statistical Analysis

All statistical analyses utilized a one-factor, repeated measures analysis of variance (ANOVA), unless otherwise specified. Where Mauchly's test indicated a violation of the sphericity assumption, the Greenhouse-Geisser correction was used. An alpha value of 0.05 was used as the criterion for statistical significance.

## Results

**Figure 4.**
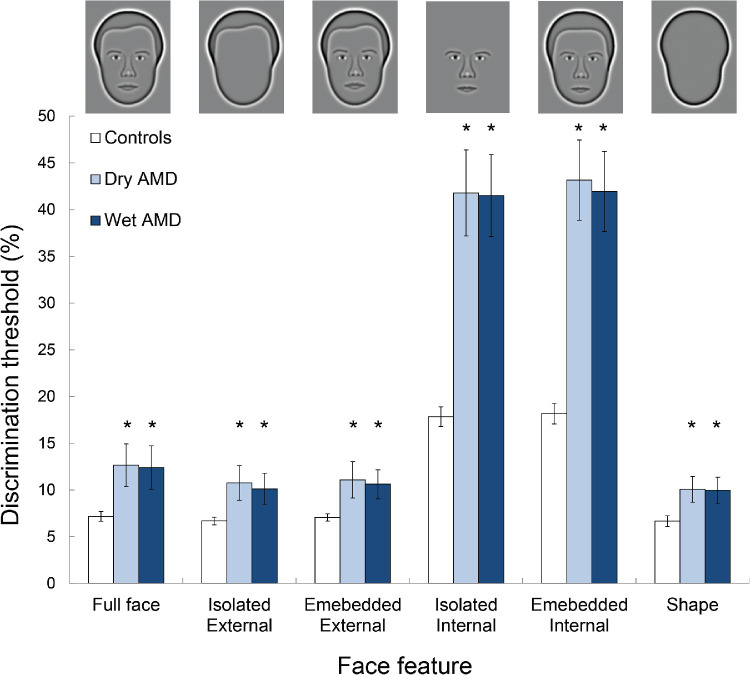
Mean discrimination thresholds for each group. Icons illustrate the feature being tested. *Error bars* represent 95% confidence intervals. *Asterisks* indicate significant difference in discrimination thresholds between AMD participants and controls (pairwise comparisons; *P* < 0.05).

Mean discrimination thresholds for each of the three groups (controls, dry AMD, and wet AMD) are given in Figure. 4. A two-factor (face feature [full faces, isolated external features, embedded external features, isolated internal features, embedded internal features and shape] and group [controls, dry AMD and wet AMD]) mixed ANOVA identified a significant main effect of group on discrimination thresholds (*F*_2,57_ = 34.81; *P* < 0.001; η_p_^2^ = 0.55). Although discrimination thresholds were significantly lower in controls relative to patients with dry (*P* < 0.001) and wet (*P* < 0.001) AMD, there was no significant difference between the thresholds associated with the two types of AMD (*P* = 0.99; pairwise comparisons with Bonferroni correction). Furthermore, there was a significant main effect of face feature on discrimination thresholds (*F*_5,285_ = 883.02; *P* < 0.001; η_p_^2^ = 0.94). The interaction between face features and group was also significant (*F*_10,285_ = 66.01; *P* < 0.001; η_p_^2^ = 0.70). Accordingly, the effect of AMD on sensitivity to each face feature was analyzed separately.

### Effect of AMD

AMD significantly reduced sensitivity to full faces, in which all of the features varied by equivalent proportions (univariate ANOVA: *F*_2,57_ = 10.03; *P* < 0.001; η_p_^2^ = 0.26). Specifically, relative to controls, full face thresholds were approximately 1.76× and 1.73× higher in patients with dry (pairwise comparisons with Bonferroni correction; *P* = 0.001) and wet (*P* = 0.001) AMD, respectively. The small difference in the magnitude of the face discrimination deficit associated with dry and wet AMD was not significant (*P* = 0.99).

A one-way analysis of covariance was carried out to investigate the effect of AMD on face discrimination ability, whilst controlling for differences in VA between controls and patients with AMD. This analysis identified a significant difference in full-face discrimination thresholds across the three groups that were tested (control participants, patients with dry AMD, and patients with wet AMD) (*F*_2,56_ = 3.28; *P* = 0.045; η_p_^2^ = 0.11). Pairwise comparisons, with Bonferroni correction, highlighted that discrimination thresholds were significantly higher, relative to those for controls, in patients with either dry (*P* = 0.042) or wet (*P* = 0.038) AMD. This result indicates that the detrimental impact of AMD on face perception cannot be solely explained by differences in VA between control participants and patients with AMD.

The same overall pattern was identified for all other face features that were tested. Specifically, AMD significantly reduced sensitivity to the external features, presented either in isolation (*F*_2,57_ = 8.52; *P* = 0.001; η_p_^2^ = 0.23) or embedded within a fixed face context (*F*_2,57_ = 8.79; *P* < 0.001; η_p_^2^ = 0.24). Further, compared to controls, discrimination thresholds for isolated (*F*_2,57_ = 52.57; *P* < 0.001; η_p_^2^ = 0.65) and embedded (*F*_2,57_ = 59.99; *P* < 0.001; η_p_^2^ = 0.68) internal features were significantly higher in patients with either type of AMD (*P* < 0.001). Type of AMD (dry or wet) had no significant effect on sensitivity to external or internal features, presented either in isolation or embedded within a fixed face context (all *P* > 0.90).

The data suggest, however, that AMD disproportionately reduces sensitivity to the internal, relative to external, features. Specifically, thresholds for the isolated external features were, on average, 1.61× and 1.51× higher, relative to controls, in patients with dry (*P* = 0.001) and wet (*P* = 0.006) AMD, respectively. On the other hand, thresholds for isolated internal features were, on average, 2.34 (*P* = 0.001) and 2.33 (*P* = 0.001) times higher in patients with dry and wet AMD. The average deficit measured in AMD patients, relative to controls, was significantly larger for the internal, compared to external, features (paired samples *t*-test; *t*_3_ = 42.658; *P* < 0.001).

Overall, AMD significantly impairs the ability to discriminate between both full faces and face features, but sensitivity to the internal features is disproportionately reduced. There was no difference between the face discrimination deficits identified in patients with dry and wet AMD.

Finally, discrimination thresholds for shapes were significantly higher in patients with AMD (Univariate ANOVA: *F*_2,57_ = 10.25; *P* < 0.001; η_p_^2^ = 0.27). Relative to controls, discrimination thresholds for shapes were 1.51× and 1.50× higher in patients with dry and wet AMD, respectively. As for faces, there was no significant effect of the type of AMD on the shape discrimination deficit (*P* = 0.99).

Figure 4 demonstrates that discrimination thresholds for features (either external or internal) presented in isolation were equivalent to those for the same features embedded within a fixed face context. This pattern was demonstrated by healthy controls, and patients with either dry or wet AMD. That the data for patients with AMD follow the same pattern as for healthy controls suggests that AMD does not lead to a qualitative change in face processing strategy.

### Association Between Low-Level Vision and Face Discrimination

**Figure 5.**
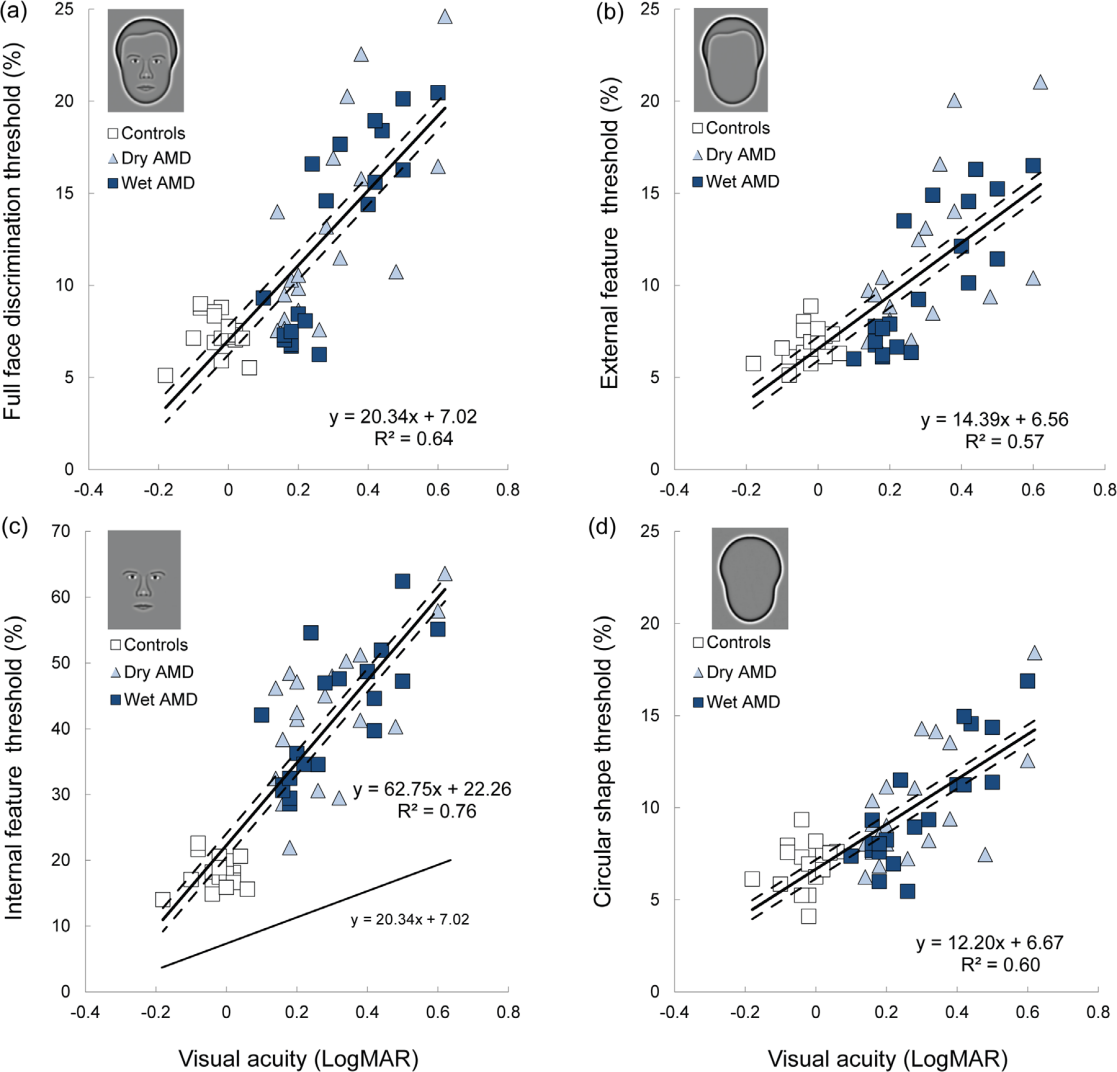
Discrimination thresholds as a function of distance VA (LogMAR) for (**a**) full faces (**b**) external features (**c**) internal features, and (**d**) shapes. *Solid line* indicates the line of best fit; *dashed lines* represent 95% confidence intervals. The external (**b**) and internal (**c**) features were presented in isolation. Thresholds for these features embedded within a fixed face context were not significantly different from those presented in isolation. Note that, because of significant differences in sensitivity across all groups, data for the internal features (**c**) are presented on a different y-axis scale. To aid visual comparison, the line of best fit for full faces, presented in (**a**), has been replicated in (**c**) for internal features (see lower line in [**c**]).

All reported relationships between VA and aspects of face perception relate to binocular, rather than monocular, VA. Regression analysis provided no evidence of a significant relationship between the binocular acuity gain (expressed as a ratio) and face discrimination thresholds, measured in patients with dry and wet AMD (*r^2^* = 0.03; F_1,38_ =1.31; *P* = 0.26). Linear regression revealed that distance VA alone accounted for 64% of the variance in full-face discrimination thresholds (*r*^2^ = 0.64; *F*_1,58_ = 103.57; *P* < 0.001). The relationship between distance VA (LogMAR) and discrimination thresholds for full faces was well captured by a linear fit (Fig. 5a). Specifically, there was a positive correlation (*r* = 0.80, *N* = 60, *P* < 0.001) between VA and full-face discrimination thresholds.

The association between VA and sensitivity to face information was analyzed separately for each feature that was tested (Fig. 5). Importantly, the external and internal feature conditions (Figs. 5b, 5c) used features which were extracted from the same faces employed within the full face condition (Fig. 5a). As a result, the effect of reduced VA in AMD on sensitivity to different face features can be directly compared.

As for full faces, there was a positive correlation (*r* = 0.75, *N* = 60, *P* < 0.001) between VA and external feature discrimination thresholds (Fig. 5b). The regression (*r*^2^ = 0.57) was significant (*F*_1,58_ = 75.32; *P* < 0.001). In the same way, there was a positive correlation (*r* = 0.87, *N* = 60, *P* < 0.001) between VA and internal feature discrimination thresholds (Fig. 5c). The regression (*r*^2^ = 0.76) was also significant (*F*_1,58_ = 188.20; *P* < 0.001). The rate of sensitivity decline with reductions in VA depended on the face feature that was tested. The regression equation for full faces had a slope of 20.34 (*t*_58_ = 10.18; *P* < 0.001). A shallower slope was identified for the external features (slope = 14.39 [*t*_58_ = 8.68; *P* < 0.001]).

The rate of decline in sensitivity to the internal features with reduced VA (slope = 62.75 [*t*_58_ = 13.72; *P* < 0.001)), on the other hand, was approximately 3.09 and 4.36 times steeper than those for full faces and external features, respectively. These results indicate that reductions in distance VA disproportionately impair sensitivity to the internal face features.

There was also a significant correlation (*r* = 0.77, *N* = 60, *P* < 0.001) between VA and shape discrimination thresholds (Fig. 5d). The regression (*r*^2^ = 0.60) was significant (*F*_1,58_ = 85.58; *P* < 0.001). The regression equation for shapes had a slope of 12.20 (*t*_58_ = 9.25; *P* < 0.001).

Similarly, linear regression revealed that contrast sensitivity alone accounted for 62% of the variance in full face discrimination thresholds (r^2^ = 0.62; F_1,58_ = 96.85; *P* < 0.001). Furthermore, standard multiple regression identified both distance VA (*t*_57_ = 2.66; *P* = 0.01) and contrast sensitivity (*t*_57_ = −2.10; *P* = 0.04) as significant independent predictors of full face discrimination thresholds (adjusted *R^2^* = 0.66; *F*_2,57_ = 57.03; *P* < 0.001) (Table 2). Combining both measurements of low-level vision explained 66% of the variance in full face discrimination thresholds. Accordingly, we estimated that sensitivity to full faces declined by a factor of approximately 1.19 ± 0.05 (mean ± 95% confidence interval) per line of VA reduction (equivalent to 0.1 LogMAR).

In sum, distinct visual functions are impaired to different extents by AMD and, owing to the common framework used in these experiments, these can be directly compared. Per unit of VA reduction, the loss in sensitivity to shapes and external face features is considerably less than that for full faces (approximately 60%) and that to full faces considerably less that to internal features (approximately 33%). Comparing sensitivity to shapes to that for internal features shows a dramatic, fivefold difference, indicating that AMD has a particularly detrimental effect on processing internal face features, which are critical to a range of everyday tasks, including interpreting facial expressions and recognizing familiar people.

**Table 2.** Standard Multiple Regression of Distance VA and Contrast Sensitivity on Full Face Discrimination Thresholds

Variables	B	SE	β	*P*
Constant	16.03	4.32		
Distance VA	11.89	4.47	0.47	0.01
Contrast sensitivity	−5.34	2.55	−0.37	0.04

## Discussion

This study used a novel test of face perception to quantify the effect of AMD on face discrimination sensitivity. Compared with age-matched control participants, patients with AMD demonstrated significant deficits in discriminating between both full faces and their component features. On average, AMD reduced sensitivity to full faces by a factor of approximately 1.75×. Interestingly, the type of AMD (dry or wet) had no impact on face discrimination ability, but the severity of the impairment was strongly correlated with measurements of low-level vision (distance VA and contrast sensitivity). Relative to controls, sensitivity to the external face features (head-shape and hairline) was on average 1.56 times poorer in AMD patients. Sensitivity to the internal features (eyes, nose, mouth, and eyebrows), on the other hand, was reduced by a factor of approximately 2.33. These data suggest that AMD does not impair discrimination of all face features equally but disproportionately reduces sensitivity to the internal features.

This study investigated the relationship between face perception and two clinical measurements of low-level vision; distance VA and contrast sensitivity. In line with previous reports, we identified a significant relationship between distance VA and sensitivity to face information in AMD,^16^^,^^17^^,^^46^ but we also established that the rate at which a reduction in VA negatively affects performance depends on the available face information.

Specific assessment of face perception is seldom undertaken in optometric clinical practice. Distance VA, however, is routinely measured by clinicians and is often utilized as a behavioral marker of AMD severity.^47^ This study supports the view that distance VA is a useful predictor of face discrimination ability in patients with AMD (distance VA explained 64% of the variance in full face discrimination thresholds). Based on our data, we estimate that sensitivity to full faces is reduced by a factor of approximately 1.19 per 0.1 (one line) of LogMAR distance VA reduction. Our data further suggest that contrast sensitivity is also a significant predictor of face discrimination ability.^20^^,^^46^^,^^48^

Although there is broad agreement that measurements of low-level vision (VA and contrast sensitivity) are related to face perception,^46^^,^^48^^,^^49^ there are some discrepancies between the findings of individual studies. Specifically, Tejeria and colleagues^46^ found no significant correlation between face recognition and contrast sensitivity in patients with AMD. It has also been proposed that reading VA is a better predictor of sensitivity to face information than distance VA.^17^^,^^50^ These discrepancies may be attributable to methodologic differences. For example, whereas this study asked participants to discriminate unfamiliar faces, the task used by Tejeria and co-workers^46^ required participants to recognize familiar faces. Furthermore, there is considerable variation in the spatial extent of the face stimuli used by individual studies. This is expected to influence the strength of the relationship between face discrimination ability and measurements of low-level vision,^16^ particularly in AMD.

The results of this study support the premise that both distance VA and contrast sensitivity are significant, independent predictors of face discrimination ability.^16^^,^^19^ Incorporating measurements of contrast sensitivity led to a significant, albeit small, increase in the proportion of variance in face discrimination thresholds explained by the regression model.

### Face Features

The present study revealed that AMD disproportionately reduces sensitivity to the internal, relative to external, face features. We interpret this result as evidence that processing of subtle differences in the shapes and positions of the internal face features is particularly vulnerable to the effects of AMD. Consistent with this premise, disruption of the retinal topography in AMD induces a perceptual distortion which reduces spatial alignment acuity.^51^^,^^52^ Furthermore, in typical participants, presenting faces in peripheral, relative to central, vision disproportionately reduces sensitivity to the internal features.^53^ This is in agreement with the proposal that impoverished spatial resolution, either in normal peripheral vision or in impaired central vision in AMD, specifically impairs discrimination of internal features. That AMD affects specific aspects of face information to greater or lesser extents argues against the premise that the detrimental impact of AMD on face perception can be explained entirely by limited spatial resolution. This is supported by our finding that significant differences in face discrimination ability persist when differences in VA between controls participants and patients with AMD are controlled.

A further important factor is crowding: impaired discrimination of visual objects due to surrounding contours.^54^ The effects of crowding become manifest when the spacing between a target object (e.g., the nose) and surrounding information (e.g., eyes and mouth) falls below a critical level.^55^ Because this critical spacing deceases with eccentricity, the effects of crowding are significantly larger in peripheral, relative to central, vision.^56^ Crowding effects are found with a wide variety of objects,^54^ including faces. Specifically, previous reports indicate that when faces are viewed within peripheral vision, the internal face features (e.g., eyes, nose, mouth) may interact to produce an intraface feature crowding effect, which substantially reduces sensitivity to face information.^57^^,^^58^

Faces typically encourage central fixation.^28^ As a result, crowding effects for control participants with healthy vision are expected to be minimal with the type of face discrimination task employed in the present study. Some patients with AMD, however, may have employed an eccentric fixation strategy in order to overcome the limitations of impaired foveal vision.^28^ Peripheral viewing of faces raises the possibility that visual sensitivity may have been limited by the effects of crowding. In support of this proposal, crowding effects significantly impair object recognition when patients with AMD employ peripheral fixation.^59^ In the present study, crowding effects may partly explain the disproportionate effect of AMD on sensitivity to the internal face features. The effects of crowing are greater when surrounding objects are visually similar.^54^ Accordingly, it seems reasonable to suggest that crowding effects will be larger with internal features (a collection of related objects comprising approximately horizontal and vertical lines) than external features (isolated global contours) (compare Fig. 3b with Fig. 3d). This is supported by the finding that sensitivity to internal features is disproportionately reduced when typical observers view faces in peripheral, rather than central, vision.^53^

It is well established that, rather than being processed as discrete parts, individual face features (e.g., eyes, nose and mouth) are integrated into an interdependent representation (i.e., holistic processing).^60^^,^^61^ Accordingly, as for global shape processing,^62^^,^^63^ disruption of retinal functioning at any point within the region of integration would be expected to degrade sensitivity to internal face features.

Sensitivity to the external features (head-shape and hairline), and shapes in general, was also impaired by AMD, albeit to a lesser extent than the internal features. Our findings are in agreement with previous studies that have reported that AMD impairs discrimination of shapes defined by deformations of a circular contour^8^ and that the severity of this impairment is correlated with distance VA.^64^

Observers rely on different visual information for different tasks. For example, the relative importance of the external and internal features depends on face familiarity. Specifically, identification of unfamiliar faces is particularly reliant on the external features.^29^^,^^65^^,^^66^ In line with this external feature advantage for unfamiliar faces, this study found that sensitivity to novel faces was considerably higher (observers better) when external, rather than internal, face information was available. This was the case for both AMD patients and controls.

Internal face features, on the other hand, make a disproportionate contribution to recognition of familiar faces.^32^^,^^33^^,^^67^ Our data show that AMD patients experience particular difficulty with recognizing faces based on information from the internal features. This suggests that AMD disproportionately affects perception of familiar faces. This is supported by reports of difficulty recognizing familiar faces from patients with AMD.^10^ The extent of AMD on sensitivity to *familiar* faces may be underestimated by this study for unfamiliar faces.

Participants with healthy vision largely fixate the internal features when viewing faces. In contrast, AMD patients direct a significantly larger proportion of their fixations toward the external face features.^28^ It may be that this atypical eye movement pattern reflects the impairment of sensitivity to internal face feature information in AMD and suggests a reason why this strategy is helpful and should be widely considered in the management of patients with AMD. Furthermore, we specifically tested whether AMD leads to a qualitative change in the strategy used to process face features. Our results, however, indicated that patients with AMD also demonstrate the same pattern of independent processing of external and internal features previously identified for participants with healthy vision.^45^

In sum, our results suggest that impaired spatial resolution and crowding significantly impair face discrimination in patients with AMD. These impairments feed-forward through the hierarchical visual system to impact on both low-level (e.g., visual acuity, contrast sensitivity) and higher-level (e.g., face and shape discrimination) aspects of visual function. Our data also indicate that certain aspects of visual function (e.g., discrimination of internal face features) are more vulnerable to the effects of AMD than others (e.g., discrimination of radial frequency patterns).

### Synthetic Faces

Previous investigations of the effect of AMD on face perception have utilized face photographs.^16^^,^^17^^,^^46^ The present study, on the other hand, employed synthetic faces which combine simplicity with sufficient realism to enable recognition of individual identities.^68^ The simplicity of these synthetic faces enables the differences between individual identities to be manipulated in a quantifiable and controlled way. This metric is highly sensitive to individual differences in face discrimination ability.^69^ Further, the essentially unlimited range of synthetic face manipulation avoids the limitations of ceiling effects with control participants, or floor effects with AMD patients, identified in previous studies with face photographs.^16^^,^^20^^,^^21^ Our previous work suggests that the synthetic face paradigm is significantly more sensitive to impairments of face perception than clinical tests based on face photographs.^69^

The synthetic face approach has some limitations. Firstly, due to their simplified nature, synthetic faces do not include all of the information available in face photographs. Synthetic faces are focused on salient face geometry (head shape, inter-ocular separation, lip thickness), other aspects of face information (e.g., hair texture, skin surface reflectance) have been excluded. The rationale for this simplification is that humans readily recognize faces over long viewing distances (e.g., 5m or more), despite significant reductions in the visibility of several aspects of face information (including hair texture and skin surface reflectance).^68^ In our view, the advantage of synthetic faces—that they can be manipulated in a quantifiable and controlled manner—outweighs the disadvantage of excluding certain aspects of face information that real faces contain but that might not be available to observers.

It should be noted that, because synthetic faces exclude both face (e.g., skin color, hair texture) and non-face (e.g., clothing, posture) information that is available in real-world contexts, the Caledonian face test may overestimate the impact of any impairment of face perception on visual functioning in a real-life context.

When using simplified stimuli, it is important to be certain that they engage the same cortical processes as more realistic stimuli. Several studies have confirmed this for synthetic faces. Synthetic faces are recognized at the individual level and across changes in viewing angle.^68^ Furthermore, synthetic faces demonstrate behavioral hallmarks of face processing, including a significant face inversion effect,^69^ external feature advantage for unfamiliar face discrimination^66^ and left-over-right visual field bias.^53^ Neuroimaging evidence indicates that synthetic faces and face photographs elicit a comparable BOLD functional Magnetic Resonance Imaging (fMRI) signal in the FFA.^70^ This suggests that the brain processes both stimuli in a similar way. Finally, patients with developmental prosopagnosia (a specific impairment of face perception) demonstrate reduced sensitivity to both face photographs and synthetic faces, but not non-face objects (e.g., cars).^69^^,^^71^

### Limitations

Due to impairment of foveal vision, AMD patients may use a peri-macular region of more peripheral retina for fixation.^72^ The location of this preferred retinal locus (PRL) varies considerably between patients and may be task-dependent.^73^^,^^74^ To avoid disadvantaging AMD patients who may use eccentric fixation, and overestimating any impairment of face discrimination, our approach did not restrict fixation and viewing time was unlimited. In the present study we did not determine the PRL or record eye movements. As a result, we could identify neither differences in gaze fixation patterns between AMD patients and controls nor the retinal area used to fixate the faces by AMD patients.

Finally, we found no effect of type (dry or wet) of AMD on sensitivity to face information. This study, however, focused on participants with relatively mild visual impairment (poorest VA = 0.62 LogMAR). It remains possible that significant differences would emerge when face perception was assessed in patients with more severe AMD.

### Conclusions

Face perception is important for social interactions and has been linked to quality of life.^9^^,^^10^ Our data indicate that patients with AMD are at risk of significant impairments of face perception. The Caledonian face test provides an objective and efficient method of assessing this important visual function which is suitable for patients with central vision loss. The quantitative assessment provided by the test may be used by clinicians to document the depth of any impairment, monitor progression and recommend rehabilitative strategies. In addition to impaired face identity discrimination, patients with AMD also demonstrate difficulty interpreting facial expressions^20^^,^^46^ and processing eye gaze direction information. Our results suggest that this is related to particular difficulty in processing internal face information in AMD.
